# Humanoid facial expressions as a tool to study human behaviour

**DOI:** 10.1038/s41598-023-45825-6

**Published:** 2024-01-02

**Authors:** G. Lombardi, A. Sciutti, F. Rea, F. Vannucci, G. Di Cesare

**Affiliations:** 1 Department of Informatics, Bioengineering, Robotics and Systems Engineering (DIBRIS), University of Genoa, Genova, Italy; 2 Cognitive Architecture for Collaborative Technologies Unit (CONTACT), Italian Institute of Technology, Genova, Italy; 3 Robotics Brain and Cognitive Sciences Unit, Italian Institute of Technology, Genova, Italy; 4 Department of Medicine and Surgery, Neuroscience Unit, University of Parma, via Volturno 39/E, 43125 Parma, Italy

**Keywords:** Neuroscience, Psychology

## Abstract

Besides action vitality forms, facial expressions represent another fundamental social cue which enables to infer the affective state of others. In the present study, we proposed the iCub robot as an interactive and controllable agent to investigate whether and how different facial expressions, associated to different action vitality forms, could modulate the motor behaviour of participants. To this purpose, we carried out a kinematic experiment in which 18 healthy participants observed video-clips of the iCub robot performing a rude or gentle request with a happy or angry facial expression. After this request, they were asked to grasp an object and pass it towards the iCub robot. Results showed that the iCub facial expressions significantly modulated participants motor response. Particularly, the observation of a happy facial expression, associated to a rude action, decreased specific kinematic parameters such as velocity, acceleration and maximum height of movement. In contrast, the observation of an angry facial expression, associated to a gentle action, increased the same kinematic parameters. Moreover, a behavioural study corroborated these findings, showing that the perception of the same action vitality form was modified when associated to a positive or negative facial expression.

## Introduction

Communication between humans comes naturally, since we are very skilled in exchanging several signals when socially interacting. Explicit signals, such as gestures, represent a rich source of information to infer goal and intentions of others’ actions. For example, if we are at a café and we observe someone moving the hand towards a cup, we can intuitively understand *what* the agent is doing (e.g. grasping the cup) and also *why* they are doing it (e.g. grasping the cup for drinking or grasping the cup for passing it towards another person). From a neurophysiological perspective, this process is due to the presence of the “mirror mechanism” based on the activity of a set of neurons (mirror neurons) located in parietal and frontal areas which discharge both when people perform goal-directed actions and when they observe other individuals performing the same actions^1–7^. However, besides goal (*what*) and intention (*why*), another fundamental aspect of the action is related to its form, i.e. *how* actions are performed. Following the previous example, according to the social context or to the mood of the agent, they can grasp the cup vigorously or delicately and pass it rudely or gently. Thus, variation in the force, direction and velocity of the agent’s action may let the observer to infer the agent’s affective state and attitude. These peculiar aspects of social communication have been defined *vitality forms* by Daniel Stern^8,9^. Recent studies demonstrated that the perception of vitality forms expressed by an agent influenced and modulated the motor response of the receiver^10,11^. Particularly, when participants perceived a rude request, regardless its modality (visual, audio, physical), their subsequent action (passing an object) was characterized by a higher velocity peak and covered a longer distance. In contrast, after a gentle request, the same action was performed with a lower velocity peak and covered a shorter distance. It is important to note that, in order to allow participants to focus their attention only on the action vitality form, these experiments excluded additional social cues such as the facial expression of the agent. However, it is well known that during social interactions, among non-verbal explicit signals, facial expressions represent one of the richest and most powerful tool from which observers can quickly and easily make inferences about emotional states^12–15^, physical health^16^, and personality traits^17^ of the agent.

From a neuroanatomical perspective, it has been recently suggested that the temporal cortex hosts two separate routes for processing static and dynamic faces^18,19^. The ventral stream for faces, including the areas V2–V4 and inferotemporal region, is involved in the recognition of the identity of individuals. The dorsal stream for faces, constituted by visual motion area MT, STS, eventually reaching pACC, anterior insula and the amygdala, is considered to be responsible for the recognition of emotions in healthy subjects.

From a behavioural point of view, emotional contagion theory has been used to explain how a facial expression conveying an emotion affects human behaviour. Specifically, people react to facial expressions seen in others tending to mimic them in their own face^20^. Additionally, some studies showed that the detection of an emotional expression induces in the observer a corresponding emotional state, thus considering the emotional contagion effect as an initial marker of affective, instead of mere motor-mimetic, reactions^21,22^.

Starting from these findings, the present study aimed to assess whether and how specific social cues such as facial expressions could modulate the motor behaviour of participants during social interactions, by proposing the iCub robot as a new tool for the investigation of this effect. Indeed, by using the iCub robot we leveraged on the possibility to manipulate positive and negative facial expressions with action vitality forms, obtaining congruent and incongruent experimental conditions. The same procedure would have been challenging and non-natural by using human actors, because the dissociation of the facial expression from the action is highly difficult to be realized (e.g. to assume a gentle attitude towards others but performing the associated gentle action with an angry face). Notably, although there are several studies regarding the interaction between humanoids and humans, the present study represents the first attempt aiming to investigate how positive or negative attitudes conveyed by the iCub robot through facial expressions and actions affect the motor response of participants.

To this purpose, we carried out a kinematic experiment in which participants observed video-clips of the iCub robot performing a rude or gentle arm action towards them (giving request) with a happy or angry facial expression. After the request, participants were asked to grasp a little ball and place it on a target as if they wanted to pass it to the iCub robot. We hypothesized that the perception of the same action performed gently or rudely could be modified when associated to a positive (happy) or negative (angry) facial expression, affecting the kinematic parameters characterizing participants motor response. Particularly, while we expected an enhancement of action vitality forms effect when the action and facial expression were congruent, no hypothesis could be provided for incongruent conditions due to their unusualness.

## Methods

### Participants

Eighteen healthy right-handed volunteers (11 females and 7 males, mean age 24.28, SD 2.42) took part in the kinematic experiment. The sample size was defined on the basis of results of an “a priori” power analysis computed with GPower 3.1 [Parameters: effect size f = 0.35; α err prob = 0.05; power (1-β err prob) = 0.9]. The output of this analysis revealed that a sample size of 16 subjects is sufficient to evidence an interaction effect between the two experimental factors (2 Facial Expressions × 2 Action Vitality Forms). All participants had normal or corrected to normal vision. None reported neurological or cognitive disorders. The study received approval by the ethical committee of Liguria Region (n.222REG2015) and was carried out according to the principles expressed in the Declaration of Helsinki. All participants provided written informed consent.

### The iCub robot and visual stimuli

In the present study, the iCub robot represented the agent of the interaction with participants. The iCub platform is a 53 degree-of-freedom humanoid robot of the same size as a 3 or 4 year-old child^23^, equipped with multiple sensors, including force/torque sensors, encoders in all its joints and eye cameras^24^. These features allow for an understanding of its body configuration, motor skills and also an ability to show facial expressions, enabling it an ideal platform for human–robot interactions studies.

**Figure 1:**
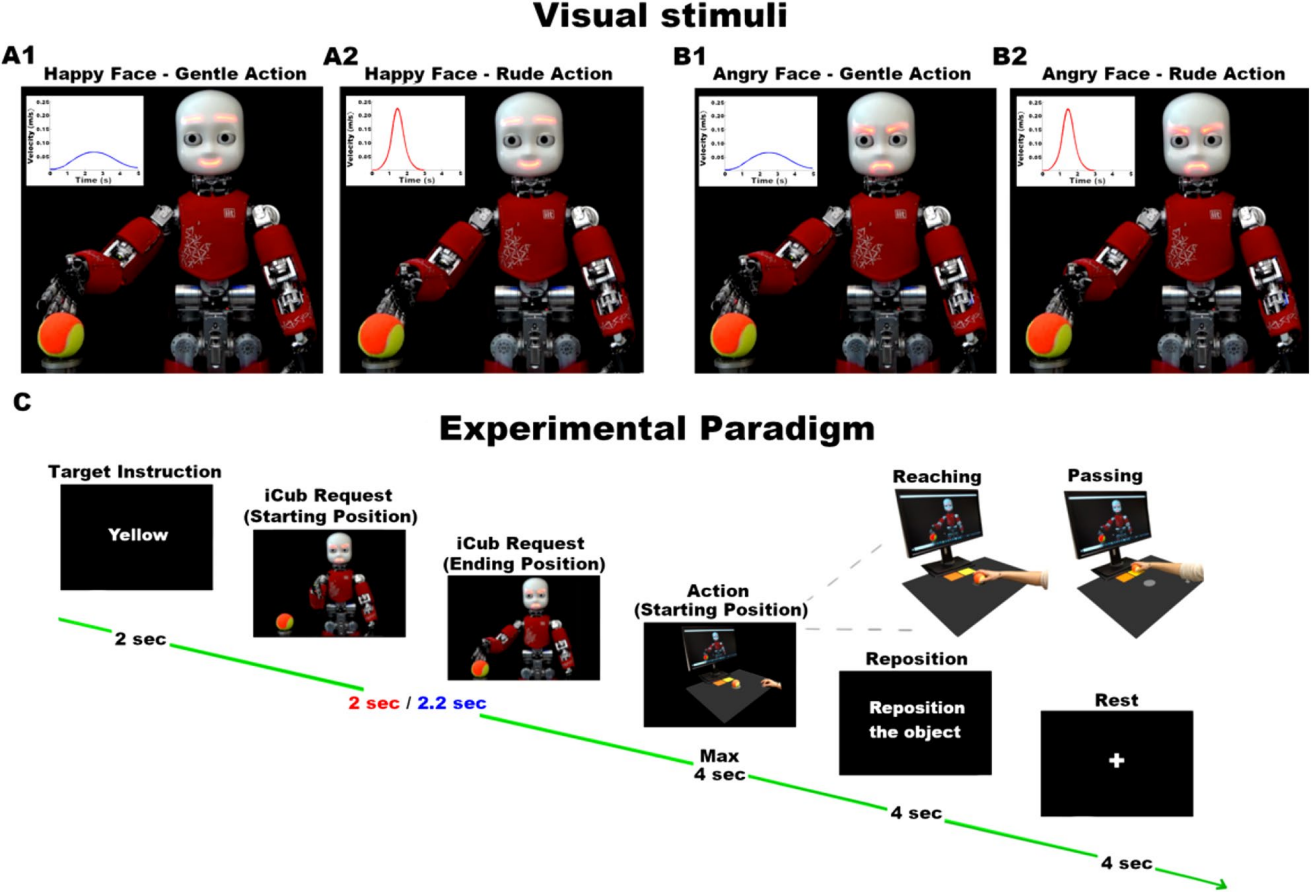
Visual stimuli representing the iCub robot giving request performed gently (blue curves, **A1**–**B1**) or rudely (red curves, **A2**–**B2**) with a happy (**A1**–**A2**) or an angry (**B1**–**B2**) facial expression. Experimental paradigm (**C**). Participants received an instruction regarding the color (Target Instruction, 2 s), observed the iCub robot request (gentle: 2.2 s, rude: 2 s) and performed the action (reaching and passing, max 4 s). Finally, they were asked to place the ball in the starting position with their left hand (Reposition, 4 s) and to wait the new trial while observing a white fixation cross on a black screen (Rest, 4 s).

Here, stimuli consisted of video-clips showing the iCub robot performing a *giving request* towards participants (Fig. 1). Specifically, the iCub robot moved its right arm gently or rudely with the palm upward inviting participants to give it a little ball. These actions have been generated by retargeting the kinematic data recorded from a trained actor, with anthropometric measures similar to those of the robot, who performed actions gently or rudely towards an object. This allowed us to replicate human actions allowing the iCub robot to perform the same requests with different vitality forms (gentle or rude). This procedure has been already used and validated in previous study^25^. In the present experiment, the iCub robot executed the request by using two different action vitality forms (gentle or rude) and showing two different facial expressions (happy or angry), for a total of four conditions. Particularly, we created two *congruent* conditions by combining facial expression and vitality form with same valence (positive: gentle action and happy face, Fig. 1A1; negative: rude action and angry face, Fig. 1B2) and two *incongruent* conditions by combining facial expression and vitality form with different valence (rude action and happy face, Fig. 1A2; gentle action and angry face, Fig. 1B1).

In order to validate these stimuli, a preliminary behavioural study was carried out (see Supplementary for details). During the kinematic experiment, stimuli were presented on a monitor in front of participants by using E-Prime software.

### Task and experimental paradigm

During the experiment, participants sat comfortably in front of a table, keeping their right hand with the thumb and index finger set in a pinching position (starting position). The starting position was located 16 cm to the right of the participant’s mid-sagittal plane and 25 cm diagonally from the centre of a circled target on which a little ball was placed. Two coloured targets (yellow and orange) were placed 20 cm from the centre of the object (Fig. 1). The monitor of a PC (25 in.) was placed closed to the coloured targets in front of participants. Participants were asked to carefully observe video-clips representing the iCub giving request and subsequently place the little ball on one of the coloured targets. To exclude the possibility that some possible spurious effects related to the participants motor behaviour (e.g. the anxiety, grasping the ball in different way etc.) could affect the experiment results, before starting the study, each participant was required to place the ball (10 consecutive trials) without receiving any request (baseline). For each participant these initial trials were then averaged to obtain the baseline condition. After the recording the baseline, participants were asked to carefully observe video-clips representing the iCub giving request and subsequently place the little ball on one of the coloured targets. Before each experimental trial, an instruction concerning the colour (yellow or orange) of the target appeared on the monitor. After the target instruction and the iCub robot request, participants had to reach and grasp the little ball and then move it (with the intention to pass it to the iCub robot) close to the monitor, placing it on the correct target (yellow or orange). After their action (reaching and passing), they returned with the right hand in the starting position. Then, an instruction on the monitor asked them to replace the little ball on the initial position with their left hand. Between two consecutive trials, a black screen with a white fixation cross was inserted as rest period (see Fig. 1C). The experiment was composed of two runs. Each run lasted about 5.30 min and consisted of 20 experimental trials presented in a randomized order. In total, 40 stimuli were shown: 20 gentle requests (10 with happy face and 10 with angry face Fig. 1A1–B1) and 20 rude requests (10 with happy face and 10 with angry face, Fig. 1A2–B2). The experiment was characterized by a 2 × 2 factorial design, with VITALITY (gentle and rude) and FACIAL EXPRESSION (happy and angry) as factors. The experiment was composed of two runs. Each run lasted about 5.30 min and consisted of 20 experimental trials presented in a randomized order. In total, 40 stimuli were shown: 20 gentle requests (10 with happy face and 10 with angry face Fig. 1A1–B1) and 20 rude requests (10 with happy face and 10 with angry face, Fig. 1A2–B2). The experiment was characterized by a 2 × 2 factorial design, with VITALITY (gentle and rude) and FACIAL EXPRESSION (happy and angry) as factors.

### Data recording

Kinematic data were acquired by using the Optitrack system V12O Trio, consisting of a self-contained and factory calibrated tracking bar positioned over the setup. A first laptop containing the Motive 2.3.4 software was used for kinematic data acquisition. A second laptop containing E-prime software was connected to the monitor positioned in front of participants and used for visual stimuli presentation. Kinematic data recording and stimuli presentation were synchronized thanks to an external sync box (Brain Products GmbH) connected between the tracking bar and the second laptop. Three passive markers were placed on the right hand of participants: the first marker on the wrist, used for the extraction of all principal kinematic data; the second and third markers on the thumb and index fingers nails respectively, used to analyse the maximum hand aperture during the reaching phase and to reconstruct possible gaps in the motion tracking recording of the first marker. The kinematic data were first pre-processed in the edit layout of Motive software and subsequently analysed with MATLAB (R2020b).

### Data analysis

During the experiment, for each participant we recorded 40 actions in response to the iCub requests (10 gentle requests with happy face, 10 gentle requests with angry face, 10 rude requests with happy face and 10 rude requests with angry face). Each motor response performed by participants was divided in two phases of interest: the reaching phase, during which participants reached the little ball and grasped it, and the passing phase, during which participants moved the little ball towards the monitor and positioned it on the requested target. For both reaching and passing phases, we extracted specific kinematic parameters: peak velocity, peak acceleration, z-coordinate trajectory (representing how much participants raised their right hand), action phase duration and time to peak velocity. For the reaching phase, we also calculated the maximum aperture of the right hand. For each participant and for each kinematic parameter, we averaged the 10 values recorded for each condition. Then we normalized these averaged values with the average of the baseline condition in which participants performed the task without receiving any request (see above). In this way, for each participant and for each kinematic parameter we obtain a single value per condition. Finally, these averaged and normalized values relative to the extracted kinematic parameters were organized to carry out a General Linear Model (GLM). The GLM considered VITALITY (gentle and rude) and FACIAL EXPRESSION (happy and angry) as two factors of interest. This model allowed us to investigate whether and how facial expressions of the iCub robot and its action vitality forms could influence the motor response of participants in terms of kinematics. All kinematic data were normalized to the baseline condition, in which participants performed the task without receiving the iCub request before. In order to investigate whether and how facial expressions and action vitality forms of the iCub robot could influence the motor response of participants, we measured possible differences of kinematic features characterizing their actions. To this aim, for each parameter, data were organized to carry out a General Linear Model (GLM), with VITALITY (gentle and rude) and FACIAL EXPRESSION (happy and angry) as two factors of interest. In addition to kinematic features, we computed the reaction time, i.e. the time elapsing between the end of the iCub request and the starting movement of participants. Also in this case, we analysed possible differences of reaction times among conditions by organizing data in a GLM with VITALITY and FACIAL EXPRESSION as factors of interest.

## Results

**Figure 2:**
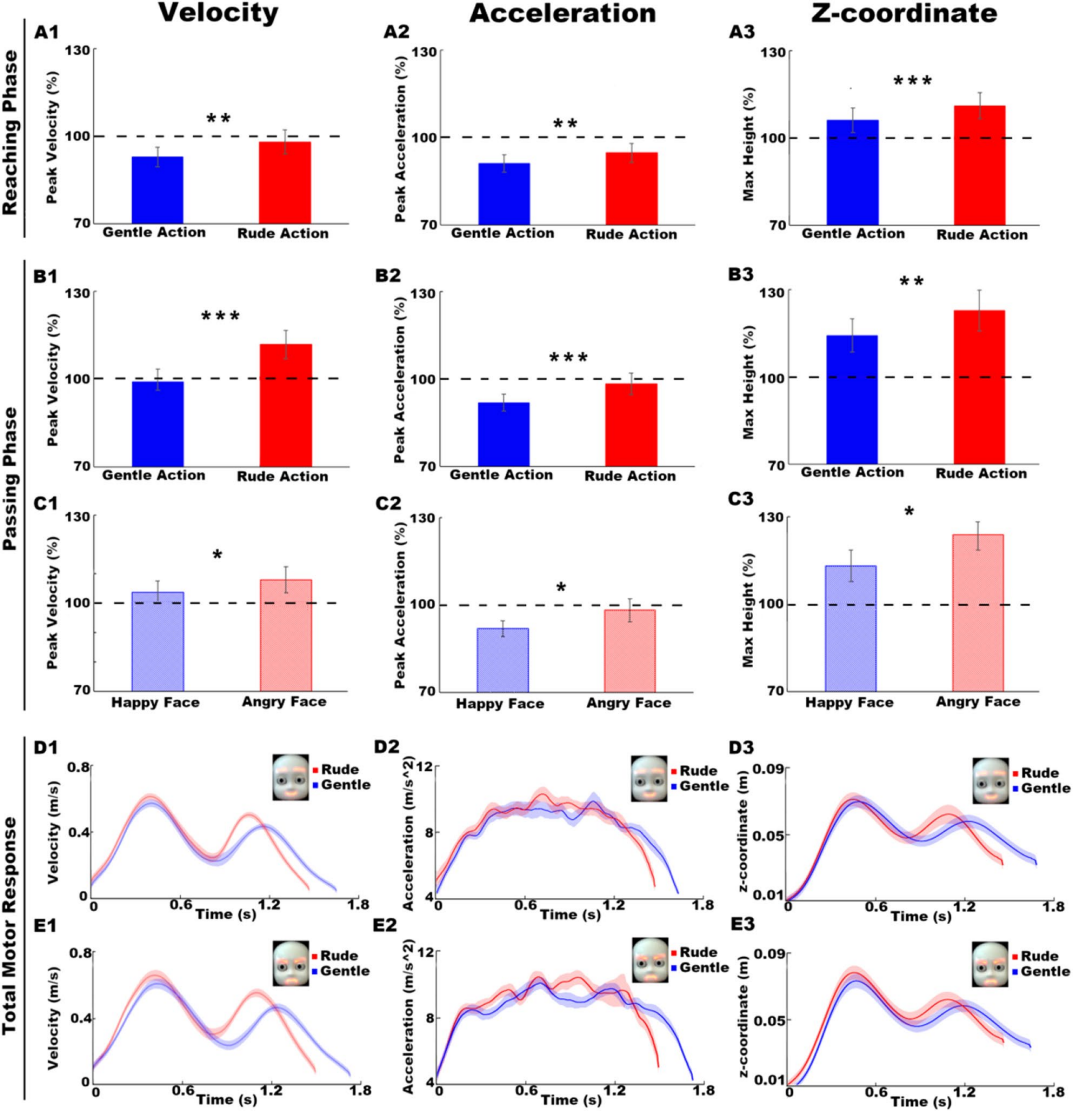
Reaching phase: main effect of VITALITY (gentle, blue bars; rude, red bars) on peak velocity (A1), peak acceleration (A2) and max height (A3). Passing phase: effect of VITALITY (gentle, blue bars; rude, red bars) on peak velocity (B1), peak acceleration (B2) and max height (B3); main effect of FACIAL EXPRESSION (happy, shaded blue; angry, shaded red) on peak velocity (C1), peak acceleration (C2) and max height (C3). The dotted line in correspondence of 100% refers to the baseline condition. Vertical bars represent the standard errors. Horizontal bars indicate statistical significance (*p ≤ 0.05, **p ≤ 0.01, ***p ≤ 0.001). Graphs below show the velocity (left panel), acceleration (middle panel) and z-coordinate (right panel) curves characterizing the total motor response of participants after gentle (blue) and rude (red) requests performed by the iCub robot with a happy (D1–D2–D3) or angry (E1–E2–E3) facial expression. Error shadings indicate standard error of the mean.

**Figure 3:**
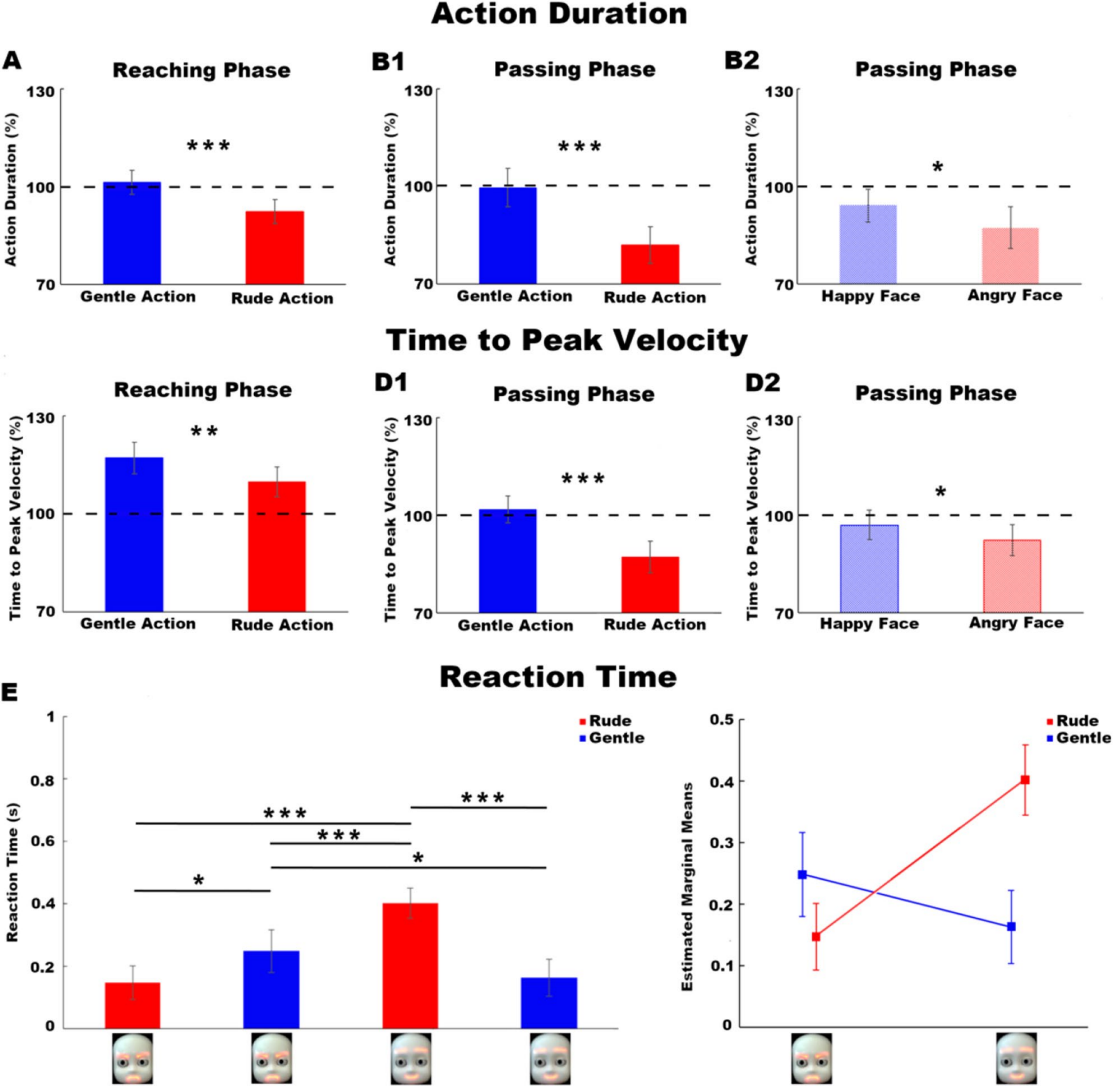
Main effect of VITALITY (gentle, blue bars; rude, red bars) on action duration (**A**: reaching phase; **B1**: passing phase) and time to peak velocity (**C**: reaching phase; **D1**: passing phase). Main effect of FACIAL EXPRESSION (happy, shaded blue; angry, shaded red) on action duration (**B2**) and time to peak velocity (**D2**) during the passing phase. The dotted line in correspondence of 100% refers to the baseline condition. Reaction time analysis (**E**). Post Hoc analysis (left panel) showing significant differences among conditions. Significant interaction VITALITY*FACIAL EXPRESSION (right panel). Vertical bars represent the standard errors. Horizontal bars indicate statistical significance (*p ≤ 0.05, **p ≤ 0.01, ***p ≤ 0.001).

The main effect of VITALITY was significant for the following kinematic parameters for both reaching and passing phases: *peak velocity* (reaching: F(1,17) = 10.43, p < 0.01 ; passing: F(1,17) = 34.32, p < 0.001; Fig. 2A1–B1), *peak acceleration* (reaching: F(1,17) = 9.26, p < 0.01; passing: F(1,17) = 14.25, p = 0.001; Fig. 2A2–B2), *max height* (reaching: F(1,17) = 15.27, p = 0.001; passing: F(1,17) = 10.93, p < 0.01; Fig. 2A3–B3), *action duration* (reaching: F(1,17) = 39.17, p < 0.001; passing: F(1,17) = 106.32, p < 0.001; Fig. 3A–B1), *time to peak velocity* (reaching: F(1,17) = 14.54, p < 0.01; passing: F(1,17) = 80.38, p < 0.001; Fig. 3C–D1). The main effect of FACIAL EXPRESSION was significant for the following kinematic parameters in the passing phase only: *peak velocity* (F(1,17) = 5.07, p < 0.05; Fig. 2C1), *peak acceleration* (F(1,17) = 5.77, p < 0.05; Fig. 2C2), *max height* (F(1,17) = 5.42, p < 0.05; Fig. 2C3), *action duration* (F(1,17) = 6.50, p < 0.05; Fig. 3B2), *time to peak velocity* (F(1,17) = 5.21, p < 0.05; Fig. 3D2). No interaction effect VITALITY*FACIAL EXPRESSION was found. Analysis of the maximum hand aperture did not reveal any significant main effect of VITALITY and FACIAL EXPRESSION. The lower part of Fig. 2 depicts the velocity (Fig. 2D1–E1), acceleration (Fig. 2D2–E2) and trajectory (z-coordinate; Fig. 2D3–E3) curves of participants actions in response to the iCub robot requests performed gently (blue curves) or rudely (red curves) with happy (Fig. 2D1–D2–D3) or angry (Fig. 2E1–E2–E3) facial expression.

Results of the *reaction time* analysis showed a significant effect of VITALITY (F(1,17) = 7.60, p < 0.05), a significant effect of FACIAL EXPRESSION (F(1,17) = 12.94, p < 0.01) and a significant interaction effect VITALITY*FACIAL EXPRESSION (F(1,17) = 51.04, p < 0.001). Post-hoc analysis (Newman–Keuls correction) revealed a significant difference of reaction times among all conditions except for the comparison of *congruent* ones (Fig. 3E).

## Discussion

The observation of actions performed by other individuals is an important source of information to understand their goal and intentions. Additionally, observing the different ways (i.e. vitality forms) in which actions are performed enables us to infer the agent attitudes and affective states. Previous studies showed that, in absence of additional social cues, gentle and rude vitality forms expressed by an agent are perceived as communicating positive and negative attitudes respectively, modulating accordingly the motor behaviour of participants^10,11^. It is noteworthy, however, that actions are nested within the social context and other non-verbal signals such as facial expressions represent a fundamental resource to understand the affective state of people we are interacting with. In this view, the present study aimed to investigate whether and how positive and negative facial expressions of an agent could modify the perception of their action vitality forms and consequently the motor behaviour of a receiver. To determine this possible effect, we tested two different conditions. Specifically, in the *congruent* condition the facial expression and the action vitality form of the agent were characterized by the same valence (positive: happy face and gentle action; negative: angry face and rude action) while in the *incongruent* condition the facial expression and the action vitality form of the agent were characterized by opposite valence (happy face and rude action; angry face and gentle action). It is important to remark that the incongruent condition was made possible thanks to the use of a humanoid such as the iCub robot. Indeed, relying on a trained actor as agent of the interaction would have been extremely challenging and not ecological during the association of two social cues (facial expression and action vitality form) characterized by opposite valence. In this regard, the use of the iCub robot as interactive and controllable agent to investigate the influence of facial expressions during social interactions represents an innovative point of our study.

Firstly, we recorded video-clips in which the iCub robot performed a rude or gentle arm action towards participants (giving request) with a happy or angry facial expression. Secondly, we validated these stimuli in a behavioural study by asking participants to describe the iCub attitude (see Supplementary for details). Results of this preliminary study indicated that, independently from the action vitality form observed (gentle or rude), the information coming from the facial expression (happy or angry) guided the choice of participants in the description of the attitude conveyed by the iCub robot. Indeed, in incongruent conditions, the valence chosen for the description of the iCub robot was the one coming from the facial expression. For example, if the iCub robot moved gently but showing an angry facial expression, participants mostly chose the adjective “angry” to describe it. These results suggested that the positive and negative facial expressions modified the perception of action vitality forms.

To confirm and deepen this hypothesis, we used the same stimuli to carry out a kinematic experiment. In this study, participants were asked to carefully observe the iCub request and subsequently to grasp a little ball and pass it towards the robot, positioning it on a target. Confirming our previous studies, results showed that vitality forms expressed by the iCub request significantly influenced the motor response of participants, during both the reaching and passing phases. Most importantly, we showed that the iCub facial expression significantly modulated several kinematic parameters characterizing the passing phase. This means that, as we hypothesized after the preliminary study, positive (happy) or negative (angry) facial expressions modified the perception of the same action vitality form (positive: gentle; negative: rude) and influenced the interaction of participants towards the robot. More specifically, if the iCub robot performed a rude request with a happy facial expression, compared to the same request with an angry facial expression, participants perceived it as communicating a positive attitude. As consequence, they interacted with the iCub robot by decreasing velocity peak, acceleration peak and maximum height of movement (Fig. 2) and by increasing action duration and time to peak velocity (Fig. 3). In contrast, if the iCub robot performed a gentle request with an angry facial expression, compared to the same request with a happy facial expression, participants perceived it as communicating a negative attitude. As consequence, they interacted with the iCub robot by increasing velocity peak, acceleration peak and maximum height of movement (Fig. 2) and by decreasing action duration and time to peak velocity.

It is important to highlight the different features characterizing the reaching and passing phases. Indeed, the reaching phase consisted of a gesture directed towards a little ball, thus requiring participants to be mostly focused on the object to correctly grasp it. For this reason, while the action vitality form of the iCub robot modulated kinematic parameters by accordingly increasing or decreasing them, the facial expression of the iCub robot did not produce a significant modulation. In contrast, the passing phase consisted of a gesture directed towards the iCub robot, agent of the interaction, thus enabling participants to naturally express their attitude towards it. In this case, besides action vitality forms, also the iCub facial expression significantly modulated kinematic parameters. This interpretation found evidence in several studies aiming to study the effect of different social contexts and intention on arm kinematics. In the late 1980s, Marteniuk et al.^26^ showed that kinematic features of participants actions were modulated when task demands required greater precision, showing that movement production is relatively specific to the constraints of the future task. This effect was also shown when participants were required to grasp an object to eat it or move it^27^ and grasp an object to lift or insert it into a niche^28^. Becchio et al. ^29^ demonstrated similar effects for social intentions, i.e. intentions directed towards another person. In this study, participants were required to move an object from a location to another (individual intention condition) or to pass it to a partner (social intention condition). Different kinematics patterns were observed for “moving” actions and “passing” actions. Moreover, Georgiou et al.^30^ showed that the kinematic features of a voluntary motor action was different in cooperative vs competitive social contexts. For example, the time movement were shorter in the competitive condition compared to the cooperative one.

Besides motor behaviour, various studies also highlighted the effect of social context on the human cognitive behaviour, showing for example that the reaction time (i.e. the time occurring between a stimulus and the initiation of a motor response) can vary depending on different social conditions^31,32^. The present belief is that two important processes take place within this reaction time: the selection of *what* needs to be achieved and *how* it needs to be achieved^33^. In this view, we aimed to understand how participants could process the different requests performed by the iCub robot and consequently change their reaction time (time occurring between the end of the iCub request and the start of their motor response). Results showed that both the facial expression and the action vitality form characterizing the iCub robot request significantly modulated the participants reaction time. Moreover, we found a significant interaction between the two factors of interest, indicating that the reaction time was remarkably affected and modulated by the perception of different action vitality forms and facial expressions. Results depicted in Fig. 3 clearly showed that participants were more rapid and spontaneous to initiate the motor response in *congruent* conditions suggesting that, when the facial expression and the action vitality form were characterized by the same valence (positive: happy face and gentle action; negative: angry face and rude action), participants easily processed this information and rapidly associated an attitude to the iCub robot. In contrast, during the *incongruent* conditions, results showed an increase of reaction times suggesting that, when the facial expression and the action vitality form had opposite valence (happy face and rude action; angry face and gentle action), participants spent more time in the cognitive processing of the attitude communicated by the iCub robot, lengthening the time between its request and their motor response. To deeply understand this effect, we carried out a subsequent behavioural study on the same group of participants. Specifically, each participant was required to observe again all the video-clips and describe their feeling towards the iCub robot by indicating an adjective from a list (see Supplementary for details). Results showed that, depending on the request, participants differently described their attitude towards the iCub robot. Notably, when the iCub robot expressed a gentle request with an angry facial expression (*incongruent* condition), participants reported to be “threatened” by it. This suggested that the negative facial expression modified the meaning typically associated to the gentle action, making the iCub robot to be perceived as a commanding agent. In line with this result, in the kinematic experiment participants increased their reaction time and decreased their velocity and trajectory during the motor response. Interestingly, when the iCub robot expressed a rude request with a happy facial expression (*incongruent* condition), participants reported to be “surprised” by it. This suggested that participants felt in a state of astonishment in response to a happy facial expression associated to a rude action, a result which easily explained the longest reaction time observed in the kinematic study during this specific condition.

Taken all together, our findings showed that, besides action vitality forms, facial expressions are essential sources of information to understand the state of an agent and thus represent a key tool for communicating with others. Most importantly, we showed that positive and negative facial expressions, associated to gentle and rude action vitality forms, can modify the perception of the action form, modulating the motor behaviour of individuals. These findings shed new light on the perception of actions during interpersonal interactions and open up new perspectives regarding some pathologies characterized by the inability to recognize emotional facial expressions. Indeed, deficits in facial emotion recognition are one of the most common cognitive impairments, and they have been extensively studied in several clinical population, including schizophrenia^34,35^, Autism Spectrum Disorder^36^, ADHD^37^, Parkinson desease^38–40^ etc. In this view, the present study is promising for future research aiming to deepen the study of affective communication in patients with face recognition impairments or as clinical method to predict the progress of a pathological condition.

### Limitations

Some potential limitations of the current study should be considered. First, during the reaching phase we did not find neither an effect of the iCub facial expression on the modulation of kinematic parameters neither an effect of the iCub action vitality form and facial expression on the maximum aperture of the grasping hand. The lack of these effects could be due to constraints related to the object type (little ball) which required a more precise grip for a correct movement. Future studies could overcome this limitation by replacing the little ball with a more easily graspable object (e.g. a bottle, a cup).

Second, participants observed a dynamic action (expression of rude and gentle vitality forms) but a static facial expression (happy and angry faces). Indeed, the iCub robot is able to convey different basic emotions with its facial expressions (e.g. happiness, anger) by simply turning on led light coming from eyebrows and mouth. In this view, the use of static facial expressions may have reduced the effect on participants’ response. Indeed, it has been recently demonstrated that, besides actions, also facial expressions can convey basic emotions with different forms. For example, joy can be expressed by a weak smiling face (low intensity) or an overt laughing (high intensity), modulating accordingly the participants’ facial reactions (emotional contagion)^41^. In this view, an interesting point for future research would be to endow the iCub robot with the ability to dynamically express facial expressions, by using different level of intensities for each basic emotion.

## Supplementary Information

Supplementary Video 1.

Supplementary Video 2.

Supplementary Information 1.

Supplementary Video 3.

Supplementary Video 4.

Supplementary Information 2.

Supplementary Information 3.

Supplementary Information 4.

## Data Availability

The datasets generated during and/or analysed during the current study are available from the corresponding author on reasonable request.
