# Author Correction: Humanoid facial expressions as a tool to study human behaviour

**DOI:** 10.1038/s41598-024-70486-4

**Published:** 2024-09-05

**Authors:** G. Lombardi, A. Sciutti, F. Rea, F. Vannucci, G. Di Cesare

**Affiliations:** 1 Department of Informatics, Bioengineering, Robotics and Systems Engineering (DIBRIS), University of Genoa, Genova, Italy; 2 Cognitive Architecture for Collaborative Technologies Unit (CONTACT), Italian Institute of Technology, Genova, Italy; 3 Robotics Brain and Cognitive Sciences Unit, Italian Institute of Technology, Genova, Italy; 4 Department of Medicine and Surgery, Neuroscience Unit, University of Parma, via Volturno 39/E, 43125 Parma, Italy

Correction to: *Scientific Reports* 10.1038/s41598-023-45825-6, published online 02 January 2024

The Acknowledgements section in the original version of this Article was omitted. The Acknowledgements section now reads:

“This work has been supported by a Starting Grant from the European Research Council (ERC) under the European Union’s Horizon 2020 research and innovation programme. G.A. No 804388, wHiSPER.”

The original Article has been corrected.

