# Supplementary material for: Humanoid facial expressions as a tool to study human behaviour
Source: Sci Rep. 2024 Jan 2;14:133. doi: 10.1038/s41598-023-45825-6 (PMC10762044; doi:10.1038/s41598-023-45825-6)
Supplement: Supplementary file 3 — Supplementary Information 1. [file 41598_2023_45825_MOESM3_ESM.pdf]

```matlab
%Experiment iCub vitality-face: kinematic analysis

clear all
close all

%Import data and find variables of interest
t=T(:,1);
x_ind=T(:,2); %index
y_ind=T(:,3);
z_ind=T(:,4);
x_poll=T(:,5); %thumb
y_poll=T(:,6);
z_poll=T(:,7);
x_polso=T(:,8); %wrist
y_polso=T(:,9);
z_polso=T(:,10);

z1=abs(z_polso-z_polso(1));
x1=abs(x_polso-x_polso(1));
y1=abs(y_polso-y_polso(1));
dt=0.0083; %frequency 120 Hz ; dt=1/120;

s=sqrt(x1.^2+y1.^2+z1.^2); %space

vx=diff(x_polso)/dt; %velocity components
vy=diff(y_polso)/dt;
vz=diff(z_polso)/dt;
v=sqrt(vx.^2 + vy.^2 + vz.^2 ); %velocity
v=smooth(v,10);

ax=diff(vx)/dt; %acceleration components
ay=diff(vy)/dt;
az=diff(vz)/dt;
a=sqrt(ax.^2 + ay.^2 + az.^2 ); %acceleration
a=smooth(a,10);

MinPeakHeight = 0.35; %decide a threshold to find peaks
MinPeakDistance = 40;

figure;hold on
findpeaks(v,'MinPeakHeight',MinPeakHeight, 'MinPeakDistance', MinPeakDistance,
'Annotate','peaks');
[pks,locs,widths,proms] = findpeaks(v, 'MinPeakHeight',MinPeakHeight,
'MinPeakDistance', MinPeakDistance);

%figure;hold on
%findpeaks(a,'MinPeakHeight',MinPeakHeight, 'MinPeakDistance', MinPeakDistance,
'Annotate','peaks');
%[pksa,locsa,widthsa,promsa] = findpeaks(a, 'MinPeakHeight',MinPeakHeight,
'MinPeakDistance', MinPeakDistance);
%hold on
%plot(v)

indexes = [];
mags = [];
duration = [];
reachIdx = [];
reachMag = [];
reachWidth = [];
```

```
returnIdx = [];
returnMag = [];
returnWidth = [];
passIdx = [];
passMag = [];
passWidth = [];

indexes = locs;
mags = pks;
duration = widths;

%reaching phase
i=1;
for j=1:length(pks)/3
   reachIdx(j,:) = indexes(i);
   reachMag(j,:) =  mags(i);
   reachWidth(j,:) = duration(i);
   i=i+3;
end

%passing phase
i=2;
for j=1:length(pks)/3
   passIdx(j,:) = indexes(i);
   passMag(j,:) =  mags(i);
   passWidth(j,:) = duration(i);
   i=i+3;
end

%returning phase
i=3;
for j=1:length(pks)/3
   returnIdx(j,:) = indexes(i);
   returnMag(j,:) =  mags(i);
   returnWidth(j,:) = duration(i);
   i=i+3;
end

reachTime = t(reachIdx);
passTime = t(passIdx);
returnTime=t(returnIdx);
reachWidth = round(reachWidth);
passWidth = round(passWidth);
returnWidth = round(returnWidth);

%First phase of interest: Reaching
reachStart = [];
reachEnd = [];
for i = 1 : length(reachIdx)
     [~, i1] = min(abs(v(reachIdx(i)+1:passIdx(i))));
     [~, i2] = min(abs(v(reachIdx(i)-reachWidth(i):reachIdx(i)-1) -
reachMag(i)*0.001));
    reachEnd = [reachEnd, i1+reachIdx(i)];
    reachStart = [reachStart, i2+reachIdx(i)-reachWidth(i)-1];
end

%Second phase of interest: Passing
passStart = [];
passEnd = [];
```

```
for i = 1 : length(passIdx)
     [~, i1] = min(abs(v(passIdx(i)+1:returnIdx(i))));
     [~, i2] = min(abs(v(passIdx(i)-passWidth(i):passIdx(i)-1) -
passMag(i)*0.001));
    passEnd = [passEnd, i1+passIdx(i)];
    passStart = [passStart, i2+passIdx(i)-passWidth(i)-1];
end

reachIdx = reachIdx.'; reachMag = reachMag.'; reachTime = reachTime.';
reachWidth = reachWidth.'; reachStart = reachStart.'; reachEnd = reachEnd.';
passStart = passStart.'; passEnd = passEnd.';
returnIdx = returnIdx.'; returnMag = returnMag.'; returnTime = returnTime.';
returnWidth = returnWidth.';
t = t.';

figure; hold on;
plot(v);
scatter(reachIdx, reachMag,'k');
scatter(passIdx, passMag,'k');
scatter(returnIdx, returnMag);
scatter(passIdx, passMag);
scatter(reachStart, v(reachStart),'r');
scatter(reachEnd, v(reachEnd),'r');
scatter(passStart, v(passStart),'g');
scatter(passEnd, v(passEnd),'g');
set(gcf, 'WindowState', 'maximized');

%Hand aperture during the reaching phase
aperture=sqrt((x_ind-x_poll).^2+(y_ind-y_poll).^2+(z_ind-z_poll).^2);
ap=zeros(20,1);
for i=1:length(pks)/3
ap(i,1)=max(aperture(reachStart(i):reachEnd(i)));
end

%Peaks acceleration reaching phase
peak_aReach=zeros(20,1);
for i=1:20
peak_aReach(i,1)=max(a(reachStart(i):reachEnd(i)));
end
%Peaks acceleration passing phase
peak_aPass=zeros(20,1);
for i=1:20
peak_aPass(i,1)=max(a(passStart(i):passEnd(i)));
end

%Max height reaching phase
max_hReach=zeros(20,1);
for i=1:length(pks)/3
max_hReach(i,1)=max(z1(reachStart(i):reachEnd(i)));
end
%Max height passing phase
max_hPass=zeros(20,1);
for i=1:length(pks)/3
max_hPass(i,1)=max(z1(passStart(i):passEnd(i)));
end

%Time
for i =1:length(ReachStart)
    for j=1:4
```

```
        t_ReachStart{i,j}=t(ReachStart{i,j});
    end
end
for i =1:length(ReachEnd)
    for j=1:4
        t_ReachEnd{i,j}=t(ReachEnd{i,j});
    end
end
for i =1:length(PassStart)
    for j=1:4
        t_PassStart{i,j}=t(PassStart{i,j});
    end
end
for i =1:length(PassEnd)
    for j=1:4
        t_PassEnd{i,j}=t(PassEnd{i,j});
    end
end

%Reaching phase time duration
for i =1:length(t_ReachEnd)
    for j=1:4
time_Reach{i,j}=t_ReachEnd{i,j}-t_ReachStart{i,j};
    end
end
%Passing phase time duration
for i =1:length(t_PassEnd)
    for j=1:4
time_Pass{i,j}=t_PassEnd{i,j}-t_PassStart{i,j};
    end
end

%Time to peak velocity reaching phase
for i =1:length(ReachPeak)
    for j=1:4
        t_ReachPeak{i,j}=t(ReachPeak{i,j});
    end
end
%Time to peak velocity passing phase
for i =1:length(PassPeak)
    for j=1:4
        t_PassPeak{i,j}=t(PassPeak{i,j});
    end
end
```
