# Supplementary figures and images for: Humanoid facial expressions as a tool to study human behaviour
Source: Sci Rep. 2024 Jan 2;14:133. doi: 10.1038/s41598-023-45825-6 (PMC10762044; doi:10.1038/s41598-023-45825-6)

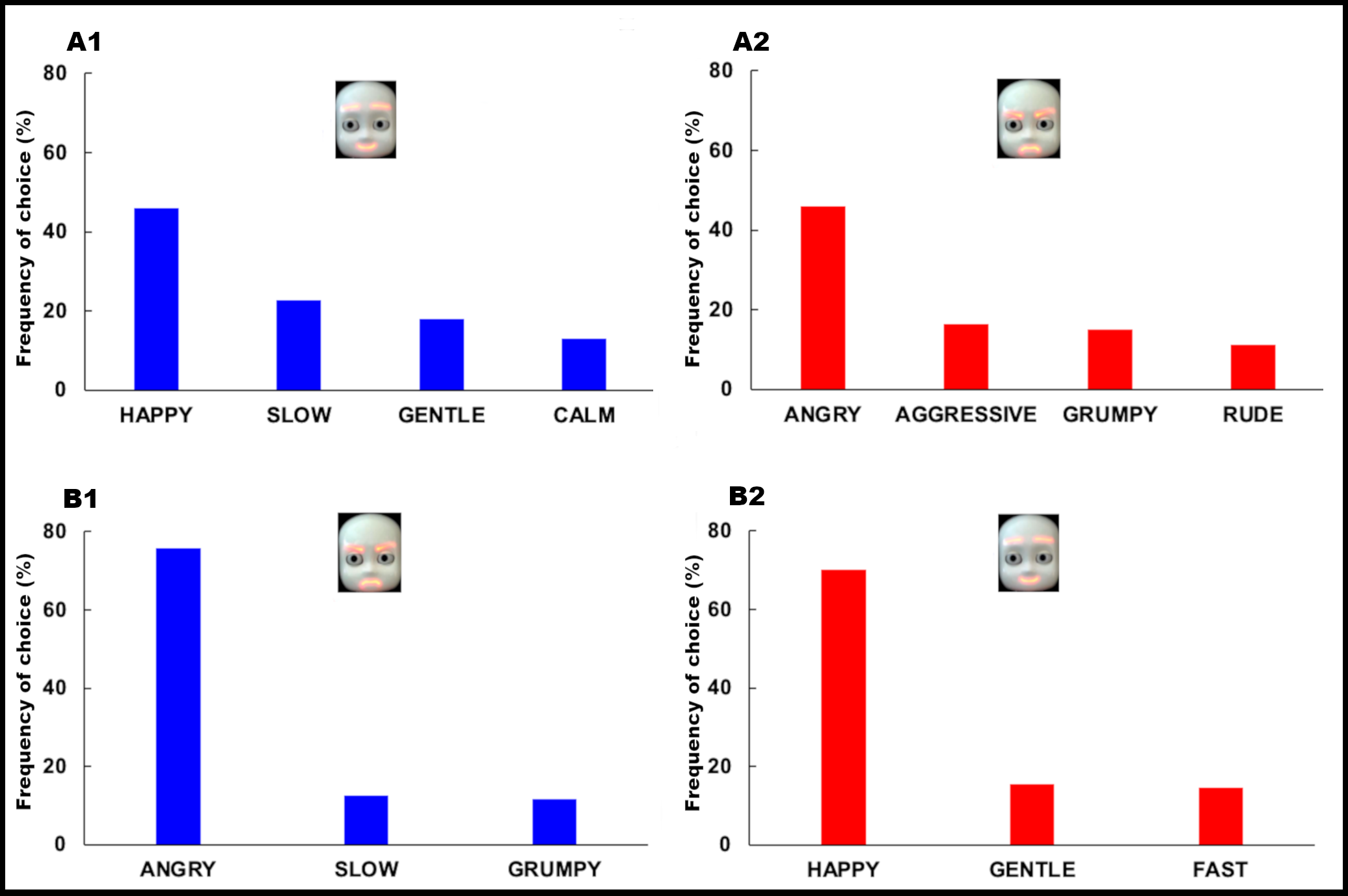

Supplement: Supplementary file 6 — Supplementary Information 2. [file 41598_2023_45825_MOESM6_ESM.png]

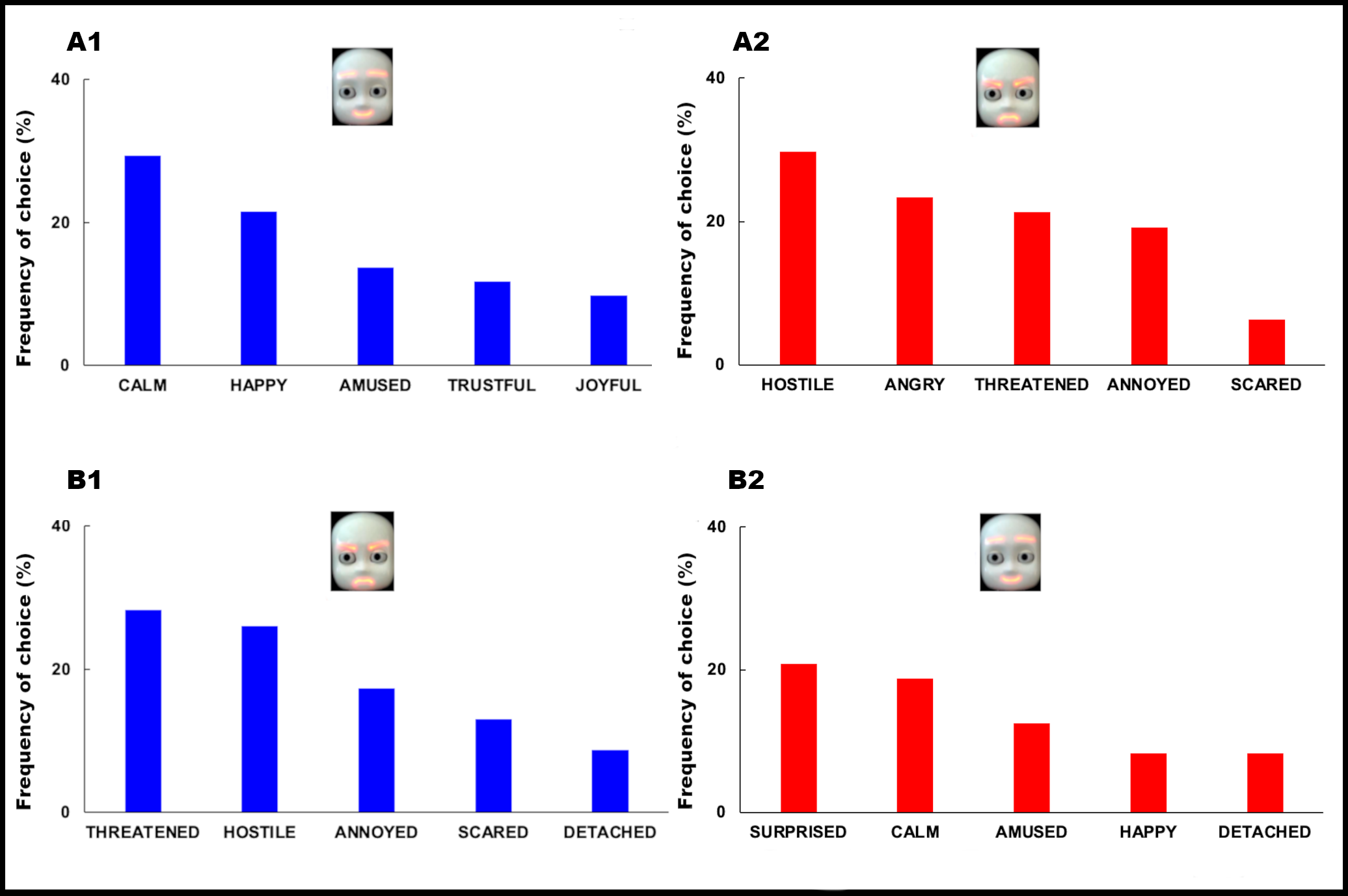

Supplement: Supplementary file 7 — Supplementary Information 3. [file 41598_2023_45825_MOESM7_ESM.png]
