# Supplementary material for: Humanoid facial expressions as a tool to study human behaviour
Source: Sci Rep. 2024 Jan 2;14:133. doi: 10.1038/s41598-023-45825-6 (PMC10762044; doi:10.1038/s41598-023-45825-6)
Supplement: Supplementary file 8 — Supplementary Information 4. [file 41598_2023_45825_MOESM8_ESM.docx]

**Supplementary Material**

*Behavioral study 1*

In order to validate visual stimuli and ascertain that all conditions were perceived as different, a preliminary behavioral study was carried out before the kinematic experiment. Forty-eight healthy participants took part in the behavioral study. Participants were presented with video-clips of the iCub robot performing an action (giving request) towards them in a gentle (GT) or rude (RD) way showing a positive (happy, HP) or negative (angry, AG) facial expression: 1) F_HP_A_GT (*congruent*); 2) F_AG_A_RD (*congruent*); 3) F_AG_A_GT: (*incongruent*); 4) F_HP_A_RD (*incongruent*). Participants were required to carefully observe each stimulus and then answer the question “how would you describe the iCub robot?” by choosing three adjectives from a given list. Considering the first choice, we decided to exclude from the statistical analysis adjectives selected with a percentage lower than or equal to 6.25%. The remaining adjectives, each one with a relative percentage of choice, were analyzed with a Chi square test. For the F_HP_A_GT congruent condition, adjectives mostly chosen were: “happy” = 46.1%; “slow” = 22.7%; “gentle” = 18.0%; “calm” = 13.3% (Figure S1 A1). The p-value obtained from the test was 3.864e-07, thus frequencies of choice were significantly different. Standardized residuals showed that “happy” was the only adjective whose frequency was greater than the expected one (5.51>|1.96|). For the F_AG_A_RD congruent condition, adjectives mostly chosen were: “angry” = 45.9%; “aggressive” = 16.5%; “grumpy” = 15.0%; “rude” = 11.3% (Figure S1 A2). The p-value obtained from the test was 1.1215e-11 thus frequencies of choice were significantly different. Standardized residuals showed that “angry” was the only adjective whose frequency was greater than the expected one (7.46>|1.96|). For the F_AG_A_GT incongruent condition, adjectives mostly chosen were: “angry” = 75.8%; “slow” = 12.5%; “grumpy” = 11.7% (Figure S1 B1). The p-value obtained from the test was 2.2e-16 thus frequencies of choice were significantly different. Standardized residuals showed that “angry” was the only adjective whose frequency was greater than the expected one (9.87>|1.96|). For the F_HP_A_RD incongruent condition, adjectives mostly chosen were: “happy” = 70.1%; “gentle” = 15.4%; “fast” = 14.5% (Figure S1 B2). The p-value obtained from the test was 3.587e-16 thus frequencies of choice were significantly different. Standardized residuals showed that “happy” was the only adjective whose frequency was greater than the expected one (8.43>|1.96|). Results indicated that, independently from the action vitality forms observed (gentle/rude), the face guided the choice of participants in the description of the iCub robot. Indeed, in conditions with incongruent valence between face and action (F_HP_A_RD; F_AG_A_GT), the valence chosen for the description of iCub was the one coming from the face. Thus, the positive and negative facial expressions modified the perception of action vitality forms. Taken these findings together, we used the same stimuli for the subsequent kinematic experiment, hypothesizing that the face of iCub would have also an
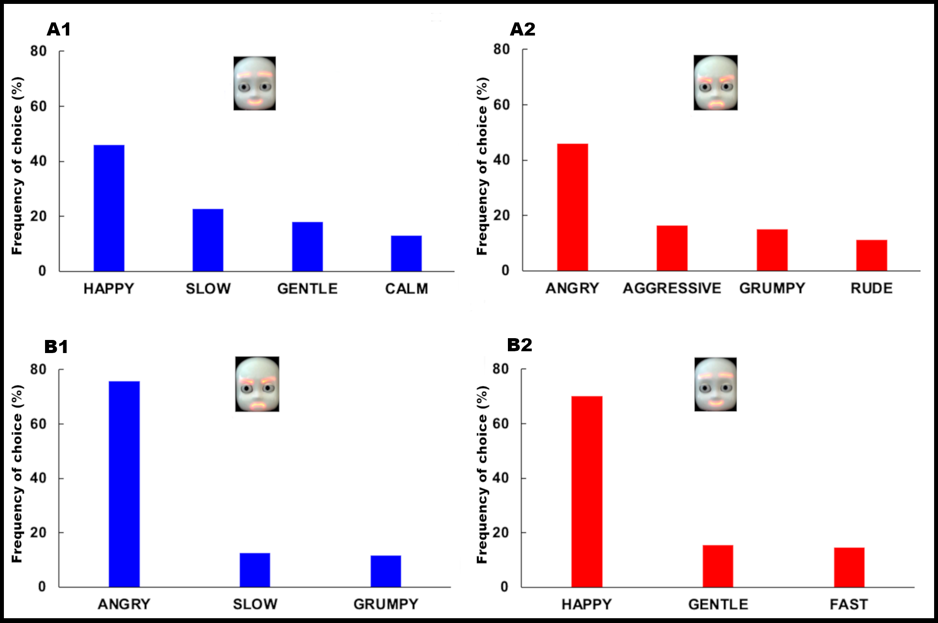
influence in the motor behavior of participants.

**Figure S1 Results of the first behavioral study. Bars represent frequency of choice of adjectives (%) used to describe each iCub robot request: A1) F_HP_A_GT congruent condition, happy face and gentle action; A2) F_AG_A_RD congruent condition, angry face and rude action; A1) F_AG_A_GT incongruent condition, angry face and gentle action; A2) F_HP_A_RD incongruent condition, happy face and rude action.**

*Behavioral study 2*

In order to deeply understand the influence of iCub request on participants affective state and motor behavior, we decided to carry out an additional behavioral study. After the kinematic experiment, each participant was required to observe again all the stimuli and answer to the question “how did you feel after this iCub request?” by choosing three adjectives from a given list. Considering the first choice, we decided to exclude from the statistical analysis adjectives selected with a percentage lower than or equal to 5%. The remaining adjectives were analyzed with a Chi square test, following the same procedure of the preliminary behavioral study described above. For the F_HP_A_GT congruent condition, the most frequently chosen adjectives were: “calm” = 29.4%; “happy” = 21.6%; “amused” = 13.7%; “trustful” = 11.8%; “joyful” = 9.8% (Figure S2 A1).. Frequencies of choice were not significantly different (p=0.13) but standardized residuals showed that “calm” had a frequency greater than the expected one (2.44>|1.96|). For the F_AG_A_RD congruent condition, adjectives mostly chosen were: “hostile” = 29.79%; “angry” = 23.4%; “threatened” = 21.28%; “annoyed” = 19.15%, “scared” = 6.38% (Figure S2 A2). Frequencies of choice were not significantly different (p=0.13) and standardized residuals did not show adjectives whose frequency was greater than the expected one. For the F_AG_A_GT condition, adjectives mostly chosen were: “threatened” = 28.26%; “hostile” = 26.1%; “annoyed” = 17.39%; “scared” = 13.04%, “detached” = 8.7% (Figure S2 B1). The p-value obtained from the test was 0.04 thus frequencies of choice were significantly different. Standardized residuals showed that “threatened” was the only adjective whose frequency was greater than the expected one (2.11>|1.96|). For the F_HP_A_RD condition, the most frequently chosen adjectives were: “surprised” = 20.83%; “calm” = 18.75%; “amused” = 12.5% followed by “happy” and “detached” with the same percentage 8.33% (Figure S2 B2). Frequencies of choice were not significantly different (p=0.2) but standardized residuals showed that “surprised” had a
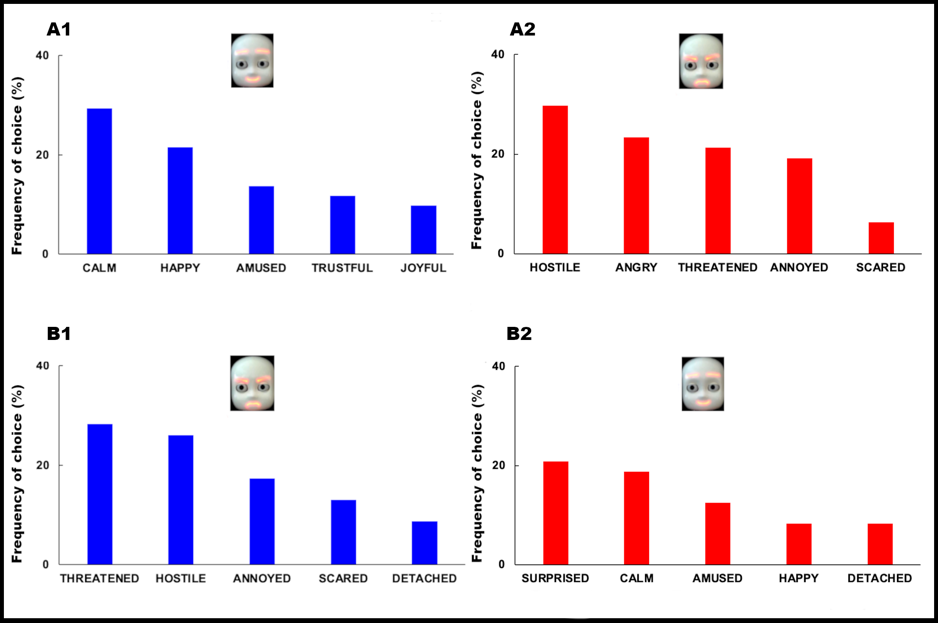
frequency greater than the expected one (2.14>|1.96|).

**Figure S2 Results of the second behavioral study. Bars represent frequency of choice of adjectives (%) which participants used to describe their attitude towards the iCub robot after each request: A1) F_HP_A_GT congruent condition, happy face and gentle action; A2) F_AG_A_RD congruent condition, angry face and rude action; A1) F_AG_A_GT incongruent condition, angry face and gentle action; A2) F_HP_A_RD incongruent condition, happy face and rude action.**
